# Book Reviews

**Published:** 1893-08

**Authors:** 


### *BOOK REVIEWS.*

---

HANDBOOK OF THE DIAGNOSIS AND TREATMENT OF THE THROAT, NOSE AND NASOPHARYNX. By Carl Seiler, M. D., Instructor in Laryngology and Lecturer on Diseases of the Upper Air Passages in the University of Pennsylvania; Chief of the Throat Dispensary at the University Hospital; Physician-in-Chief of the Union Dispensary, etc.

This is one of the most useful works to the student, the general practitioner and the specialist which has been produced by any one in this line in a long time. As a guide to the practitioner and a text-book for the student, it is unexcelled, being plain, accurate, comprehensive, and pleasantly written. The clever author has lost no pains in making himself thoroughly understood. He takes up the subjects of instruments, pathology, diagnosis and treatment, and with that full knowledge acquired only by long experience, makes them so clear that no one can fail to comprehend. It is a work which should adorn the library of every student of the medical art.

CHOLERA: ITS CAUSES, SYMPTOMS, PATHOLOGY AND TREATMENT. By Roberts Bartholow, M. D., LL. D., Emeritus Professor of Materia Medica, General Therapeutics and Hygiene in the Jefferson Medical College of Philadelphia. Cloth, $1.25. Philadelphia: Lea Brothers & Co., 1893.

This volume of one hundred and twenty-seven pages is an excellent description of the etiology, symptoms and management of epidemic cholera. The introduction gives briefly the history of the epidemics that have visited this country during the last sixty years. In the chapter on Etiology the author gives the relation of climate, elevation, and water supply to outbreaks of the disease; also a full description of the cholera bacillus in its etiological relations. The chapter on Treatment, both prophylactic and actual, is specially complete and modern. Means of disinfection and quarantine are discussed and different methods of treatment outlined. These include use of acids, antiseptics, enteroclysis, intravenous infusion, hypodermatoclysis, Rumpf's treatment (calomel) at Hamburg, the treatment at the Paris hospitals, at the New York quarantine, etc.

---

HUMAN ANATOMY. A Complete Systematic Treatise by Various Authors. Edited by Henry Morris, M. A. and M. B., London, Surgeon to, and Lecturer on Surgery, formerly Lecturer on Anatomy at the Middlesex Hospital; Late Examiner in Anatomy at the University of Durham, etc. Philadelphia: P. Blakiston, Son & Co., 1012 Walnut street.

The subject of Anatomy has been well covered for years with excellent text-books, and the one before us will certainly add another. This volume is the first attempt, so far as we know, to have the different divisions of Anatomy prepared by different authors, and this can only be regarded as a wise innovation. The section on Osteology has been written by Mr. Bland Sutton, the one on Joints by the editor, the one on Muscles by Mr. J. H. Davis-Colley, the one on Blood Vessels by Mr. W. J. Walsham, the one on the Urinary and Generative Organs by Mr. William Anderson, etc. The section on the Nervous System, prepared by Dr. St. John Brooks, is one of the most thorough in the whole work; so also is the section on Surgical and Topographical Anatomy, prepared by

Mr. W. H. A. Jacobson, the last being especially practical and helpful. In these two divisions of the work the authors have made every allowance for late additions to our knowledge on such matters as Cranial Topography, Localization of Cerebral and Spinal Functions, etc.

One great merit of this work is that so many of the illustrations are reproductions of actual dissections done by master hands. Anatomy cannot be taught by diagrams. It cannot be taught very well by photographs, but photographs of actual dissections are immensely superior to the diagrammatic representations of the artist. We note some changes in names, which are improvements. The *supinator longus*, which, by the way, is said to be not a supinator at all, is called the *brachio* (or *humero*) *radialis*. The *extensor primi internodii pollicis* is called the *extensor brevis pollicis*, and the *extensor secundi internodii pollicis* the *extensor longus pollicis*, etc. The work contains too many errors. There are some not noticed in the table of errata. In the plate on page 529 the inferior thyroid artery is called the thyroid axis. The plate on page 610 shows no external popliteal nerve. (It is a little curious that Gray's Anatomy makes the same omission, page 604.) Most of these errors are of little consequence, and are doubtless as few as could be expected in the first edition of such a large work. The subject described gives little opportunity for originality, but the old, old story of human anatomy is retold in a manner which is above unkind criticism. There are seven hundred and ninety-one good illustrations, many of which are taken from dissections. It is a work of great merit, and we expect to see it take a prominent place among standard text-books of its class. We note that it is already recommended in many medical colleges.

